# Use of computed tomography and automated software for quantitative analysis of the vasculature of patients with pulmonary hypertension

**DOI:** 10.1590/0100-3984.2016.0163

**Published:** 2017

**Authors:** Danilo Tadao Wada, Adriana Ignácio de Pádua, Moyses Oliveira Lima Filho, José Antonio Marin Neto, Jorge Elias Júnior, José Baddini-Martinez, Marcel Koenigkam Santos

**Affiliations:** 1 MSc, Attending Physician at the Centro de Ciências das Imagens e Física Médica (CCIFM) of the Hospital das Clínicas da Faculdade de Medicina de Ribeirão Preto da Universidade de São Paulo (HCFMRP-USP), Ribeirão Preto, SP, Brazil; 2 PhD, Attending Physician in the Pulmonology Department of the Hospital das Clínicas da Faculdade de Medicina de Ribeirão Preto da Universidade de São Paulo (HCFMRP-USP), Ribeirão Preto, SP, Brazil; 3 PhD, Attending Physician in the Cardiology Department of the Hospital das Clínicas da Faculdade de Medicina de Ribeirão Preto da Universidade de São Paulo (HCFMRP-USP), Ribeirão Preto, SP, Brazil; 4 PhD, Professor in the Department of Internal Medicine of the Hospital das Clínicas da Faculdade de Medicina de Ribeirão Preto da Universidade de São Paulo (HCFMRP-USP), Ribeirão Preto, SP, Brazil; 5 PhD, Collaborating Professor in the Department of Internal Medicine of the Hospital das Clínicas da Faculdade de Medicina de Ribeirão Preto da Universidade de São Paulo (HCFMRP-USP), Ribeirão Preto, SP, Brazil

**Keywords:** Hypertension, pulmonary, Tomography, X-ray computed, Image processing, computer-assisted, Hipertensão pulmonar, Tomografia computadorizada, Processamento de imagem assistida por computador

## Abstract

**Objective:**

To perform a quantitative analysis of the lung parenchyma and pulmonary vasculature of patients with pulmonary hypertension (PH) on computed tomography angiography (CTA) images, using automated software.

**Materials and Methods:**

We retrospectively analyzed the CTA findings and clinical records of 45 patients with PH (17 males and 28 females), in comparison with a control group of 20 healthy individuals (7 males and 13 females); the mean age differed significantly between the two groups (53 ± 14.7 vs. 35 ± 9.6 years; *p* = 0.0001).

**Results:**

The automated analysis showed that, in comparison with the controls, the patients with PH showed lower 10th percentile values for lung density, higher vascular volumes in the right upper lung lobe, and higher vascular volume ratios between the upper and lower lobes. In our quantitative analysis, we found no differences among the various PH subgroups. We inferred that a difference in the 10th percentile values indicates areas of hypovolemia in patients with PH and that a difference in pulmonary vascular volumes indicates redistribution of the pulmonary vasculature and an increase in pulmonary vasculature resistance.

**Conclusion:**

Automated analysis of pulmonary vessels on CTA images revealed alterations and could represent an objective diagnostic tool for the evaluation of patients with PH.

## Abstract (pt)

Resumo

**Objetivo:**

Avaliar, quantitativamente, as alterações do parênquima e da vasculatura pulmonar em exames de angiotomografia computadorizada (angio-TC) de pacientes com hipertensão pulmonar (HP), utilizando programa de análise totalmente automática.

**Materiais e Métodos:**

Avaliamos, retrospectivamente, prontuários e exames de angio-TC de 45 pacientes com HP (28 mulheres e 17 homens; idade de 53 ± 14,7 anos), em comparação com um grupo controle mais jovem sem comorbidades detectadas (13 mulheres e 7 homens; idade de 35 ± 9,6 anos). Os grupos diferiram estatisticamente quanto à idade (*p* = 0,0001).

**Resultados:**

Análise automática mostrou valores menores do percentil 10 da densidade dos pulmões, maior volume vascular do lobo superior direito e volume vascular relativo entre lobos superiores e inferiores nos pacientes com HP, quando comparados com o grupo controle. Não encontramos diferenças da análise quantitativa por angio-TC entre grupos de pacientes com HP. Inferimos que a diferença nos valores do percentil 10 indique a presença das áreas de oligoemia nos pacientes com HP, enquanto as diferenças nos volumes vasculares indiquem redistribuição da trama e aumento da resistência vascular pulmonar.

**Conclusão:**

A análise quantitativa automática da vasculatura pulmonar em imagens de angio-TC identificou alterações e pode representar ferramenta diagnóstica objetiva na avaliação da HP.

## INTRODUCTION

Pulmonary hypertension (PH) is a chronic clinical disease characterized by elevated pulmonary vascular resistance and pressure associated with extensive vascular proliferation and remodeling. It is defined as a mean pulmonary artery pressure (mPAP) greater than or equal to 25 mmHg at rest or greater than 30 mmHg during exercise^(1)^, as determined by right heart catheterization (RHC). The PH classification most widely used in clinical practice is the 2013 Nice classification system^(1-3)^: group 1 comprises forms of pulmonary arterial hypertension, including idiopathic forms; group 2 comprises forms of PH secondary to disease of the left heart; group 3 comprises forms of PH secondary to chronic lung parenchyma disease or chronic hypoxia; group 4 comprises forms of PH secondary to chronic thromboembolism; and group 5 comprises forms of PH secondary to poorly understood multifactorial mechanisms.

Despite the advances in noninvasive imaging methods, RHC with mPAP measurement remains the gold standard for the diagnosis of PH. Despite the low risk of adverse events, catheterization is an invasive diagnostic method and should not be performed without an appropriate indication^(4)^. Various methods and tools have been used in attempts to find a replacement for catheterization in the diagnosis of PH or to reduce the number of indications for RHC^(5-8)^. However, only a few of those methods have been incorporated in the clinical routine for the evaluation of patients with PH, one such method being computed tomography (CT) of the chest, especially CT angiography (CTA) of the pulmonary arteries.

New tools for quantitative and functional evaluation have been used in imaging studies to increase the diagnostic capacity of the methods, as well as to provide information that is more objective and has prognostic value. Computerized quantitative analysis of chest CT images has been used in the evaluation of various lung diseases, mainly emphysema, as well as airway diseases (such as asthma and cystic fibrosis) and interstitial lung diseases. The method has been used in order to describe the natural progression of the diseases, assess severity, stratify the prognostic risk, and monitor the treatment, showing a good correlation with the pathological findings and functional test results^(9-11)^.

The objective of this study was to perform a quantitative analysis of the parenchyma and pulmonary vasculature on CTA images of patients diagnosed with PH by RHC. To that end, we used a computer program with fully automated analysis capability.

## MATERIALS AND METHODS

### Patients

This study was approved by the Research Ethics Committee of the Hospital das Clínicas da Faculdade de Medicina de Ribeirão Preto da Universidade de São Paulo (HCFMRP-USP). Because it was a retrospective study based on examinations already performed by patients with a clinical indication for the follow-up/evaluation of PH, the need for informed consent was waived.

**Figure 1:**
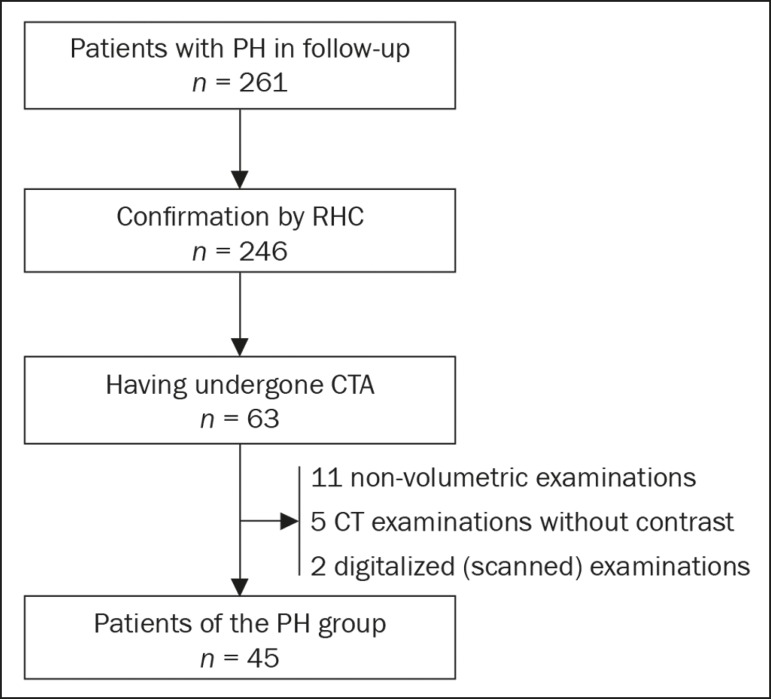
Algorithm of the inclusion and exclusion criteria applied in the selection of the PH group patients.

We retrospectively assessed the physical, electronic, and CTA records of adult patients with PH in clinical follow-up at our reference hospital. The RHC and CT examinations were requested as part of the routine clinical evaluation of these patients, there being no description of investigation of infection or suspicion of another acute complication. We included patients who had undergone CTA at our facility on the same devices and with confirmation by RHC with mPAP measurement. Patients for whom the technical quality of the tests was considered inadequate for diagnosis and quantitative analysis were excluded. After we applied the selection criteria, the sample comprised 45 patients with PH (Figure 1).

The control group comprised 20 examinations of young patients submitted to CTA for investigation of acute pulmonary thromboembolism, with a negative result, without other clinical or laboratory signs of pulmonary thromboembolism or other pulmonary vascular disease. In this group, we excluded individuals with radiological or clinical signs of diffuse lung disease, focal lesions greater than 3.0 cm, heart disease, or other significant changes detectable by CTA.

### CT of the chest

CT scans were performed on multidetector devices with inspiratory volumetric images obtained after intravenous administration of iodinated contrast medium in a single bolus injection followed by a saline flush. Other typical parameters are as follows: slice thickness ≤ 2 mm; reconstruction interval ≤ 1 mm; voltage, 120 kVp; current, 150-200 mAs; gantry rotation, 0.3-0.7 s. The volumetric acquisitions were reconstructed with soft and hard filters, with windows for the mediastinum and lung, and analyzed on a dedicated workstation.

### Qualitative evaluation by CT

We evaluated the examinations qualitatively with the Horos free image viewer, version 1.1.6 for Macintosh, obtaining the measurements of the pulmonary trunk and ascending aorta, as well as the pulmonary trunk/ascending aorta ratio. We also evaluated dilation/dilatation or hypertrophy of the right ventricle, contrast medium reflux into the hepatic veins, attenuation of the lung parenchyma (homogeneous, mosaic, or ground-glass centrilobular nodules), and pulmonary opacities resulting from infarction (bands, streaks, consolidations, and others).

### Quantitative analysis

Quantitative analysis of CT images was performed with the academic program Yacta, version 2.6^(12)^. The Yacta program was developed by a group of researchers affiliated with the University of Heidelberg, in Germany, and is used at HCFMRP-USP thanks to a research partnership between the two institutions.

**Figure 2:**
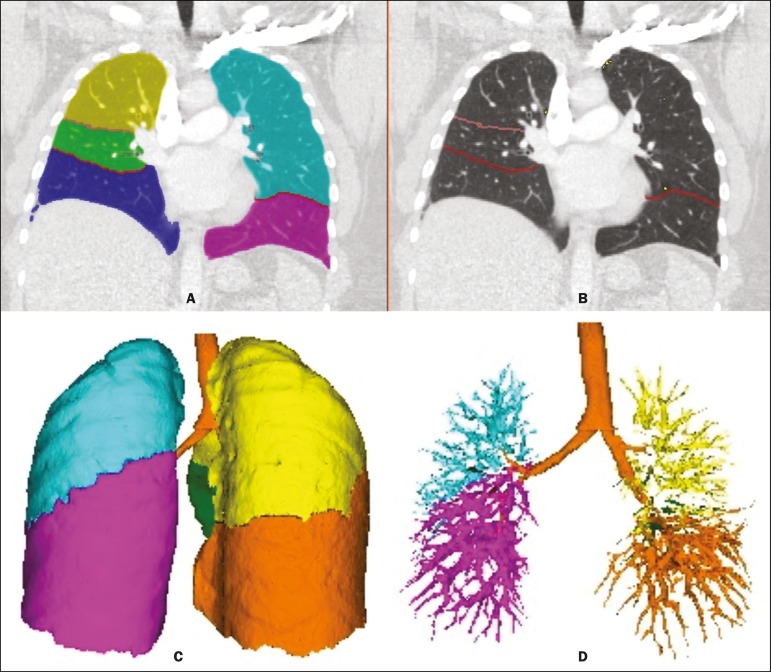
Illustrative reconstructions of the automated pulmonary segmentation performed by the Yacta automated program. **A:** Coronal reconstruction of the lung, each lung lobe represented by a different color (yellow = RUL, green = middle lobe, dark blue = RLL, light blue = LUL, and pink = LLL). **B:** Coronal reconstruction with pulmonary fissures delimited by red lines and pulmonary emphysema detected represented by yellow color pixels. **C:** Volumetric rendering image illustrating the trachea and each lung lobe segmented by the program. **D:** Volumetric rendering of the airways and pulmonary vasculature of each segmented lobe.

The Yacta program works completely automatically, requiring no user intervention at any stage of the process (Figure 2). The analysis of the images takes 4-9 min after they have been sent for processing. Initially, Yacta segments (anatomically separates) the airways, blood vessels, lungs, and lung lobes; it then supplies the lung volumes and densities, together with the volume of blood vessels in each lung lobe. The program uses an attenuation coefficient of −500 HU as the standard threshold for the detection of vessels and then segments them according to the lobar division already performed for the airways. In lungs with an altered attenuation coefficient, the program calculates a new threshold on the basis of an attenuation coefficient histogram. Intrapulmonary voxels with coefficients above the calculated threshold are then labeled as vessels, and vessels with three-dimensional communication larger than 100 mm^3^ are counted. Smaller voxels are rejected. The value obtained is then multiplied by a correction factor related to the body size of the individual. In addition to the lung volumes and mean densities, the program provides the relative values of lung parenchyma density, including the percentiles. In this study, we arbitrarily selected the 10th percentile (p10) of lung densities in an attempt to differentiate between the PH and control patients, in analogy to the correlation shown in quantitative studies of emphysema with the 15th percentile (p15)^(13)^.

### Statistical analysis

All data were organized and analyzed on a personal computer, with the spreadsheet program Microsoft Excel 2011 and the statistical analysis program Medcalc, version 9.4 (MedCalc Software, Mariakerke, Belgium). The Shapiro-Wilk test of normality was used in order to verify the normal distribution of the variables. Unpaired *t*-tests were used for comparison between the PH and control patients, as well as between PH subgroups 1 and 4, with a significance level of 95% (*p* < 0.05). For the most significant variables, we also evaluated the receiver operating characteristic (ROC) curve for the PH diagnostic test and the Pearson correlation index for the correlation with the mPAP values.

## RESULTS

We evaluated 45 patients with PH (28 females and 17 males; mean age, 53 ± 14.7 years) and 20 control subjects (13 females and 7 males; mean age, 35 ± 9.6 years). Patients with PH were also divided according to the Nice classification: subgroup 1 (*n* = 24); subgroup 2 (*n* = 2); subgroup 3 (*n* = 2); and subgroup 4 (*n* = 17). Subgroup 1 was composed of 20 females and 4 males, with a mean age of 44 ± 16 years, whereas subgroup 4 was composed of 11 females and 6 males, with a mean age of 54 ± 10 years. . The mean age was significantly higher in subgroup 4 than in subgroup 1 (*p* = 0.025).

**Figure 3:**
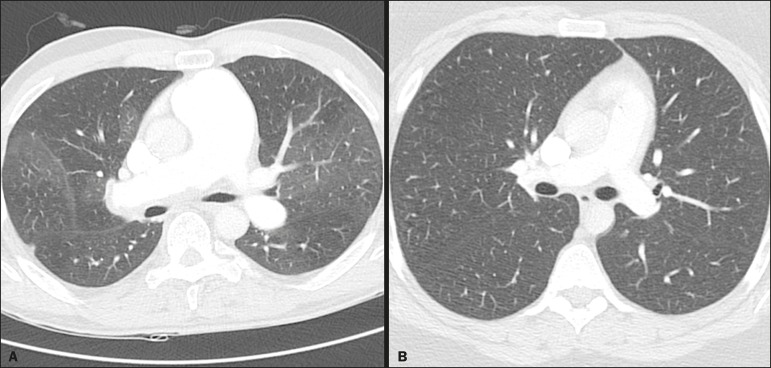
Axial CT images showing the difference in pulmonary attenuation between a patient with PH (**A**) and a patient of the control group (**B**). In **A**, we can observe areas of low attenuation (oligemia) in the lung parenchyma of a patient with PH, with a quantitative value of p10 of lung density of −873 HU, as obtained by the Yacta program. The image in **B** shows homogeneous attenuation of the lung parenchyma of a patient of the control group (p10: −760 HU).

We found no significant differences between the PH and control patients in terms of the mean pulmonary volumes and densities. However, the quantitative CT analysis of the lung parenchyma showed a significant difference in the p10 for lung density, the values being lower in the PH patients (Table 1 and Figure 3).

**Table 1:** Main parameters of automated quantitative analysis performed on CTA images of PH patients and control patients, as well of the patients in the most important PH subgroups (subgroup 1 - pulmonary arterial hypertension; subgroup 4 - chronic thromboembolism).

Group	Pulmonary volume (cm^3^)	Pulmonary density (HU)	p10 for pulmonary density (HU)	Pulmonary vascular volume (cm^3^)	Vascular density (x 10^-3^)
Control	2,987 ± 741	-652 ± 78	-799 ± 59	86 ± 21	29.7 ± 6.4
PH	3,067 ± 1,142	-668 ± 85	-829 ± 54 *	90 ± 31	29.9 ± 6.9
Subgroup 1	3,042 ± 1,382	-667 ± 85	-824 ± 64	85 ± 35	29.3 ± 8.0
Subgroup 4	3,232 ± 723	-680 ± 39	-840 ± 32	95 ± 15	28.77 ± 3.7

p10, 10th percentile; Vascular density = vascular volume / pulmonary volume.

* Statistically different (p < 0.05).

**Figure 4:**
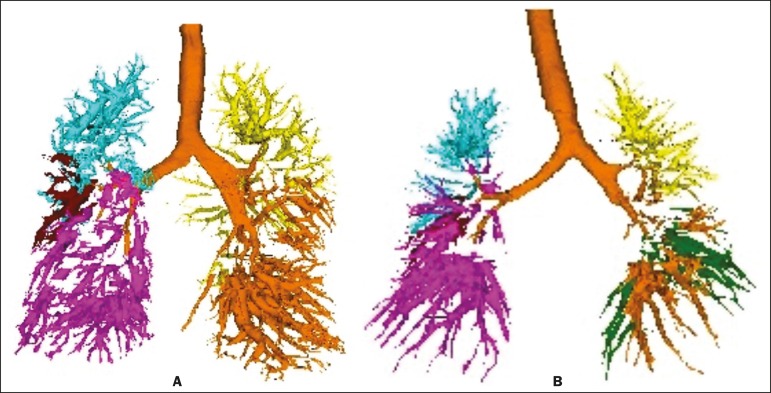
Three-dimensional reconstruction of the pulmonary vasculature by the Yacta automated program. The image in **A** shows the relatively greater volume of the pulmonary vasculature in the upper lobes than in the lower lobes in a patient with PH (ULs/LLs = 1.692), whereas the image in **B** shows a different picture in a patient of the control group (ULs/LLs = 0.6486).

There was no significant difference between the PH and control patients in terms of the total pulmonary vascular volume, although the vascular volume of the right upper lobe (RUL) was higher in the PH patients than in the control patients. This difference was not significant for the left upper lobe (LUL) or for the right and left lower lobes (RLL and LLL, respectively). In the analysis of the relative vascular volume values, considering the ratio between the upper and lower lobes (ULs/LLs), we found higher values in the PH patients than in the control patients (*p* = 0.0006), as well as for the RUL/RLL and LUL/LLL ratios (Table 2 and Figure 4). In the PH patients, the vascular volume was 17% higher in the upper lobes than in the lower lobes (ULs/LLs ratio, 1.17). In the control patients, the vascular volume in the upper lobes was only 54% of that obtained for the lower lobes (ULs/LLs ratio, 0.54).

**Table 2:** Parameters of the automated quantitative analysis performed on CTA images of PH patients and control patients, considering the lobar distribution of vessels.

Group	RUL vessels (cm^3^)	LUL vessels (cm^3^)	RUL/RLL vascular volume ratio	LUL/LLL vascular volume ratio	ULs/LLs vascular volume ratio
Control	13.3 ± 5.8	13.3 ± 5.1	0.5472 ± 0.1889	0.5712 ± 0.2412	0.5436 ± 0.1529
PH	18.5 ± 9.3*	16.5 ± 8.3	1.0825 ± 1.0992*	2.2274 ± 5.1819*	1.1731 ± 1.0915*

RUL, right upper lobe; LUL, left upper lobe; RLL, right lower lobe; LLL, left lower lobe; ULs = upper lobes; LLs, lower lobes

* Statistically different in comparison with the control group (p < 0,05).

In the quantitative CT analysis, we found no significant difference between the two largest subgroups of PH patients (subgroup 1 and subgroup 4).

**Figure 5:**
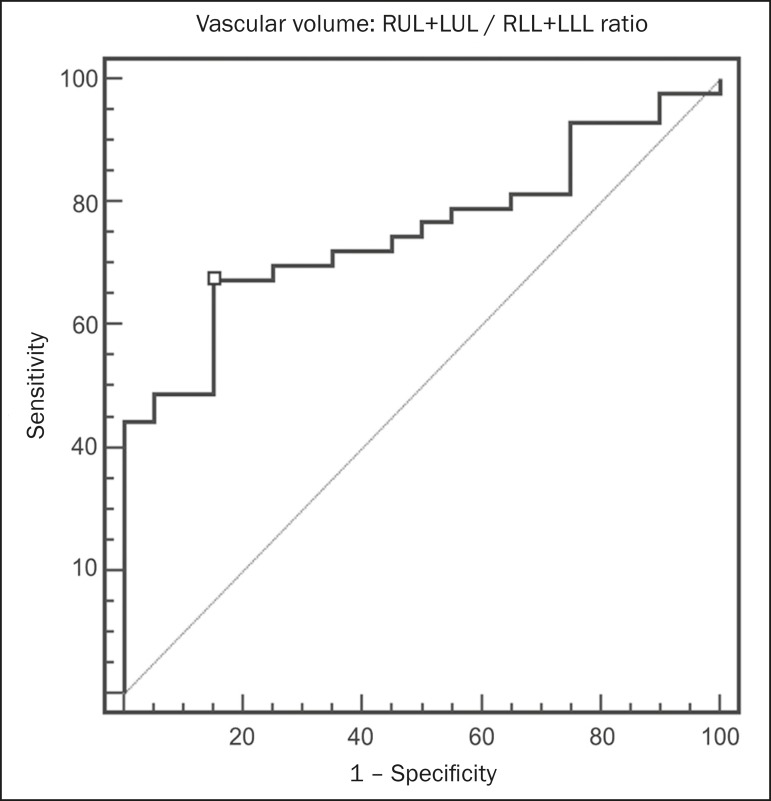
ROC curve for the quantitative CT measurement of the ULs/LLs ratio.

For the variable with the most significant difference between the PH patients and the control patients-the ULs/LLs ratio-we analyzed the ROC curve. This analysis showed an area under the curve of 0.753, the most accurate cut-off ULs/LLs ratio being 0.64 (64%), with a sensitivity of 67.4% and a specificity of 84.1% (Figure 5). Using the Pearson correlation index, we found no significant correlation between the quantitative CT variables and the mPAP values (ρ < 0.3), even for the measures that presented the most significant difference in comparison with the control group values.

The data obtained in the qualitative analysis of the PH patients were consistent with data in the literature. The most common finding was dilatation of the pulmonary trunk with simultaneous dilatation of the segmental arteries and right ventricle, which was seen in 95% of the patients, followed by contrast medium reflux into the hepatic veins, seen in 70%, right ventricular hypertrophy, seen in 60%, and mosaic attenuation of the lung parenchyma, seen in 45%. Ground-glass centrilobular nodules were seen in 13% of the PH patients (*n* = 6; 5 in subgroup 1). 

We identified one isolated case of pulmonary trunk dilatation in a patient in the control group.

## DISCUSSION

In this study, the quantitative analysis of the CTA images of PH patients was performed using a fully automated program. The volume of the RUL vessels and the ULs/LLs ratio were higher in the PH patients than in the control patients. We infer that this finding probably represents the redistribution of the pulmonary vasculature, which pathologically indicates an increase in pulmonary vascular resistance. We also found that the p10 values for the mean density of the lung parenchyma were lower in the PH patients. This finding is likely representative of the presence of areas of low attenuation, indicating oligemia related to the pulmonary vascular disease. These quantitative measures, obtained in CTA examinations routinely used in the clinical evaluation of PH patients, show potential as objective, reproducible tool for the diagnosis, prognosis, and follow-up of patients with pulmonary vascular disease.

Despite all of the advances in imaging, the most widely used measure for the investigation of suspected PH is the diameter of the pulmonary trunk and its relationship with the ascending aorta measured in the CT^(14,15)^. However, some studies^(16,17)^ have questioned the specificity of the most commonly proposed pulmonary trunk diameter cut-off value, which is 29 mm^(14-21)^. It should be borne in mind that normal pulmonary trunk diameters are frequently found in cases of mild PH, and a normal value therefore does not rule out the diagnosis. The diameter of the pulmonary artery relative to that of the ascending aorta at the same level has been suggested as a more specific measure in moderate and severe cases of PH^(15)^. Dilatation and tortuosity of the segmental pulmonary arteries (with a diameter 1.25 times that of the adjacent bronchus) in at least three lobes, in the absence of significant lung parenchymal disease, accompanied by dilatation of the pulmonary artery trunk, has also been described as a finding with high specificity^(22)^.

The technological evolution of CT devices and computer analysis programs allowed the development of objective, quantitative tools for analysis of the lung parenchyma (in cases of emphysema or fibrosis), the airways (in cases of chronic obstructive pulmonary disease, asthma, or cystic fibrosis), and, more recently, the pulmonary vessels. Some studies, such as that conducted by Ando et al.^(23)^, have demonstrated the potential of quantitative evaluation of the pulmonary vasculature for the detection of the effect of treatment with vasodilators in patients with chronic obstructive pulmonary disease and PH.

In this study, the quantitative parameter obtained in CT images with the greatest statistical significance was the ULs/LLs ratio, which was higher in the PH patients. We infer that this finding likely represents redistribution of the vasculature and an increase in pulmonary vascular resistance. The pulmonary vascular bed is a system of low resistance, and, when in the upright position, vessels supplying the upper lobes are smaller and fewer in comparison with those in the lung base^(24)^. In the supine position this difference tends to disappear, and the evaluation of the pulmonary vasculature has traditionally been given little weight in CT examinations. Our study demonstrates, in an objective way, that alteration of the pulmonary vasculature in patients with PH can be appreciated even when the examination is performed with the patient in the supine position.

Another interesting finding of this study was the difference between the PH and control patients in the p10 value for mean pulmonary density. The p10 value is a relative measure that is related to heterogeneity of the lung parenchyma and the presence of areas of low attenuation. A similar measure is that of the 15th percentile of pulmonary densities, suggested, for example, for the characterization of pulmonary emphysema as an alternative to the most widely used threshold of −950 HU^(25-27)^. Although mosaic attenuation is an isolated finding with little specificity in the context of pulmonary vascular diseases, it is known that the pathological areas are those of low attenuation, which represent focal hypoperfusion of the parenchyma, associated with vascular oligemia^(25,28)^. We infer that the difference in the p10 values found in this study represents the presence of such areas of low attenuation in the lung parenchyma, associated with hypoperfusion/oligemia. Therefore, the quantification of those areas also has potential in the imaging characterization of pulmonary vascular disease.

This study has limitations. Although the ULs/LLs ratio differentiated the PH patients from the control patients, we did not find a good correlation between the ULs/LLs ratio and the mPAP measurements. One possible explanation for this is that our group of PH patients was heterogeneous, with a predominance of individuals in subgroups 1 and 4. It is possible that individualized studies of each of the PH subgroups would produce different results, given that the subgroups differ in terms of the pathophysiological mechanism underlying the development of PH and the clinical course of the disease. The fact that the CTA and the RHC examinations were performed on different dates might also have contributed to this limitation. In addition, not all RHC examinations were performed at the same facility and with the same equipment. Despite the advanced algorithm applied by the automated program, in approximately one third of the cases there were problems related to the automated lobar segmentation, mainly in terms of the identification of the middle lobe and the lingula. In such cases, those segments were incorporated into the upper lobes or the lobar evaluation was not included in the statistical analysis, only the analysis of the right and left lungs being maintained. We cannot unequivocally rule out the possibility that subjects in the control group had undiagnosed underlying cardiac or lung disease at the time of evaluation, especially because none of those patients underwent RHC. Finally, in our study, it was not possible to index the quantitative values obtained for weight and height, because the relevant data were missing from some medical records, especially those of the control patients. Indexing those values could have increased the accuracy of the comparative analysis between the groups^(29)^.

## CONCLUSION

Quantitative analysis of the pulmonary vasculature and of attenuation of the lung parenchyma seen on CTA can provide objective data on PH and potentially on other pulmonary vascular diseases. These quantitative measures obtained automatically in examinations already used in the clinical routine of patient evaluation have potential as an objective, reproducible tool for the diagnostic evaluation, grading, and follow-up of patients with PH or other diseases that affect the pulmonary vasculature.
